# Supplementary material for: Addressing the Challenges and Barriers to the Integration of Machine Learning into Clinical Practice: An Innovative Method to Hybrid Human–Machine Intelligence
Source: Sensors (Basel). 2022 Oct 29;22(21):8313. doi: 10.3390/s22218313 (PMC9653746; doi:10.3390/s22218313)
Supplement: Supplementary file 1 [file sensors-22-08313-s001.zip › sensors-1975440-supplementary.pdf]

Addressing the challenges and barriers to the integration of machine learning into clinical practice: An innovative method to hybrid human-machine intelligence

Supplementary Materials

***Table S1**, Descriptive statistics of the quantitative data*

	mean	std	min	25%	50%	75%	max
**ONSYRS**	6.497403e+00	6.363143	0.00	2.000000e+00	4.000000e+00	9.000000e+00	40.000
**AGEATONSET**	3.011948e+01	9.259797	8.00	2.300000e+01	2.900000e+01	3.600000e+01	54.000
**DIAGYRS**	3.567532e+00	4.745402	0.00	1.000000e+00	1.000000e+00	5.000000e+00	40.000
**AGEATDIAGYRS**	3.304935e+01	9.513807	13.00	2.600000e+01	3.300000e+01	4.000000e+01	58.000
**T2LESIONSCREENING**	4.965065e+01	36.846517	0.00	2.225000e+01	4.200000e+01	7.100000e+01	249.000
**NormalBrainVolume**	1.580130e+06	96188.787187	1278789.06	1.518173e+06	1.586498e+06	1.649965e+06	1851174.430
**T2VOLUMESCREENING**	9.861367e+00	11.662986	0.00	1.960500e+00	5.734500e+00	1.348200e+01	99.054
**GADOSCREENING**	1.370130e+00	3.652584	0.00	0.000000e+00	0.000000e+00	1.000000e+00	38.000
**GDVOLUMESCREENING**	1.822369e-01	0.509063	0.00	0.000000e+00	0.000000e+00	1.230000e-01	5.019
**T1LESIONSCREENING**	2.725714e+01	27.619208	0.00	7.000000e+00	1.800000e+01	4.100000e+01	204.000
**T1VOLUMESCREENING**	3.007651e+00	4.771851	0.00	3.157500e-01	1.125000e+00	3.732000e+00	39.201
**RLPS1YP**	1.571429e+00	0.675189	1.00	1.000000e+00	1.000000e+00	2.000000e+00	5.000
**EDSSSCREENING**	2.442857e+00	1.207629	0.00	1.500000e+00	2.000000e+00	3.500000e+00	5.500
**BOWLBLDRSCREENING**	4.415584e-01	0.642927	0.00	0.000000e+00	0.000000e+00	1.000000e+00	3.000
**BRAINSTMSCREENING**	5.818182e-01	0.766110	0.00	0.000000e+00	0.000000e+00	1.000000e+00	4.000
**CEREBELRSCREENING**	1.022078e+00	0.976062	0.00	0.000000e+00	1.000000e+00	2.000000e+00	4.000
**CEREBRALSCREENING**	4.324675e-01	0.673662	0.00	0.000000e+00	0.000000e+00	1.000000e+00	2.000
**DISTWALKSCREENING**	4.861039e+02	53.137108	150.00	5.000000e+02	5.000000e+02	5.000000e+02	500.000
**PYRAMIDLSCREENING**	1.579221e+00	1.031158	0.00	1.000000e+00	1.000000e+00	2.000000e+00	5.000
**SENSORYSCREENING**	8.675325e-01	0.975972	0.00	0.000000e+00	1.000000e+00	2.000000e+00	4.000
**VISUALSCREENING**	5.948052e-01	0.859113	0.00	0.000000e+00	0.000000e+00	1.000000e+00	4.000

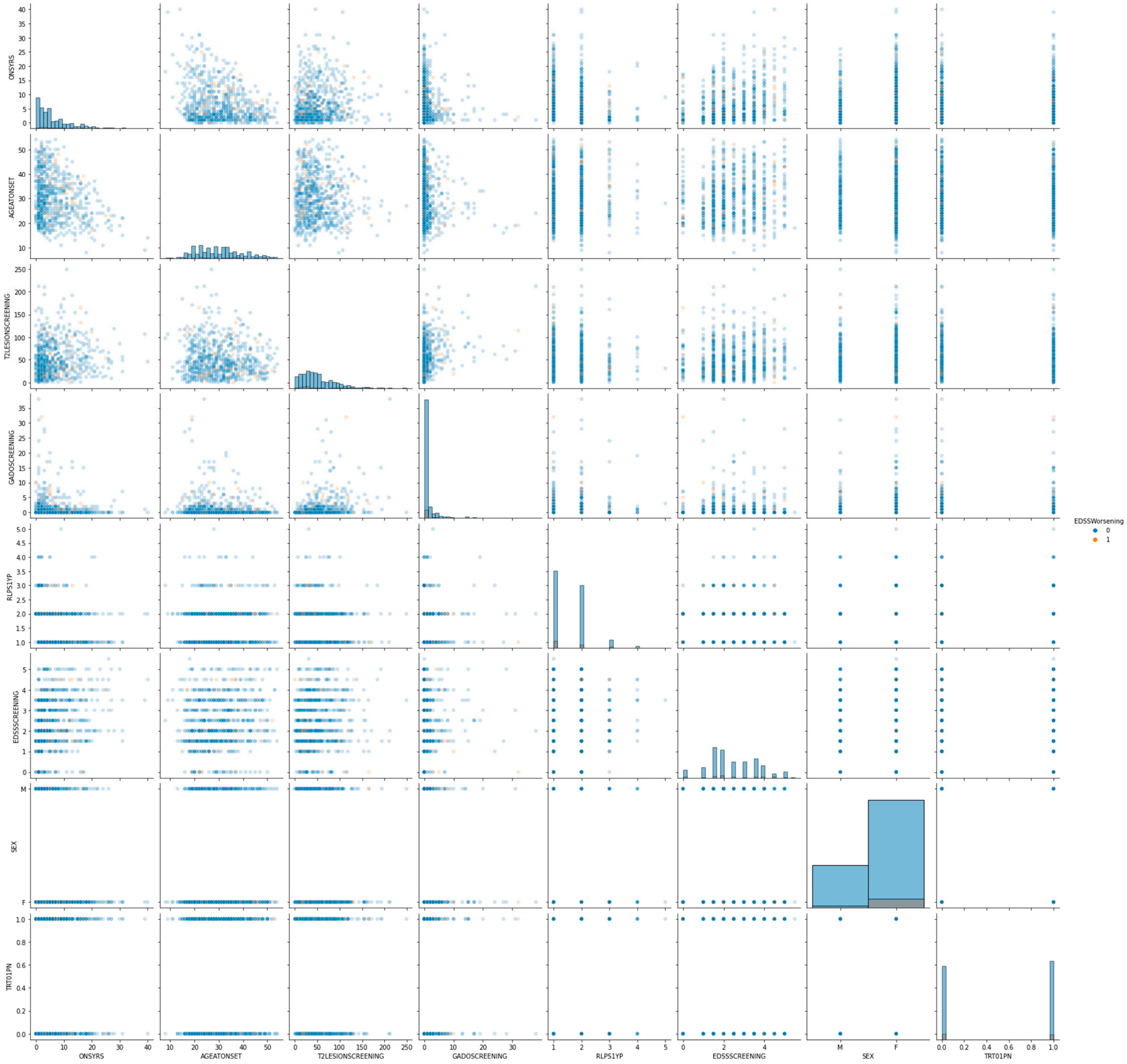

Figure S1, *Pairwise graph showing how the features used by the physician differ by EDSS worsening. The diagonal shows the distribution of individual variables for each EDSS worsening class*

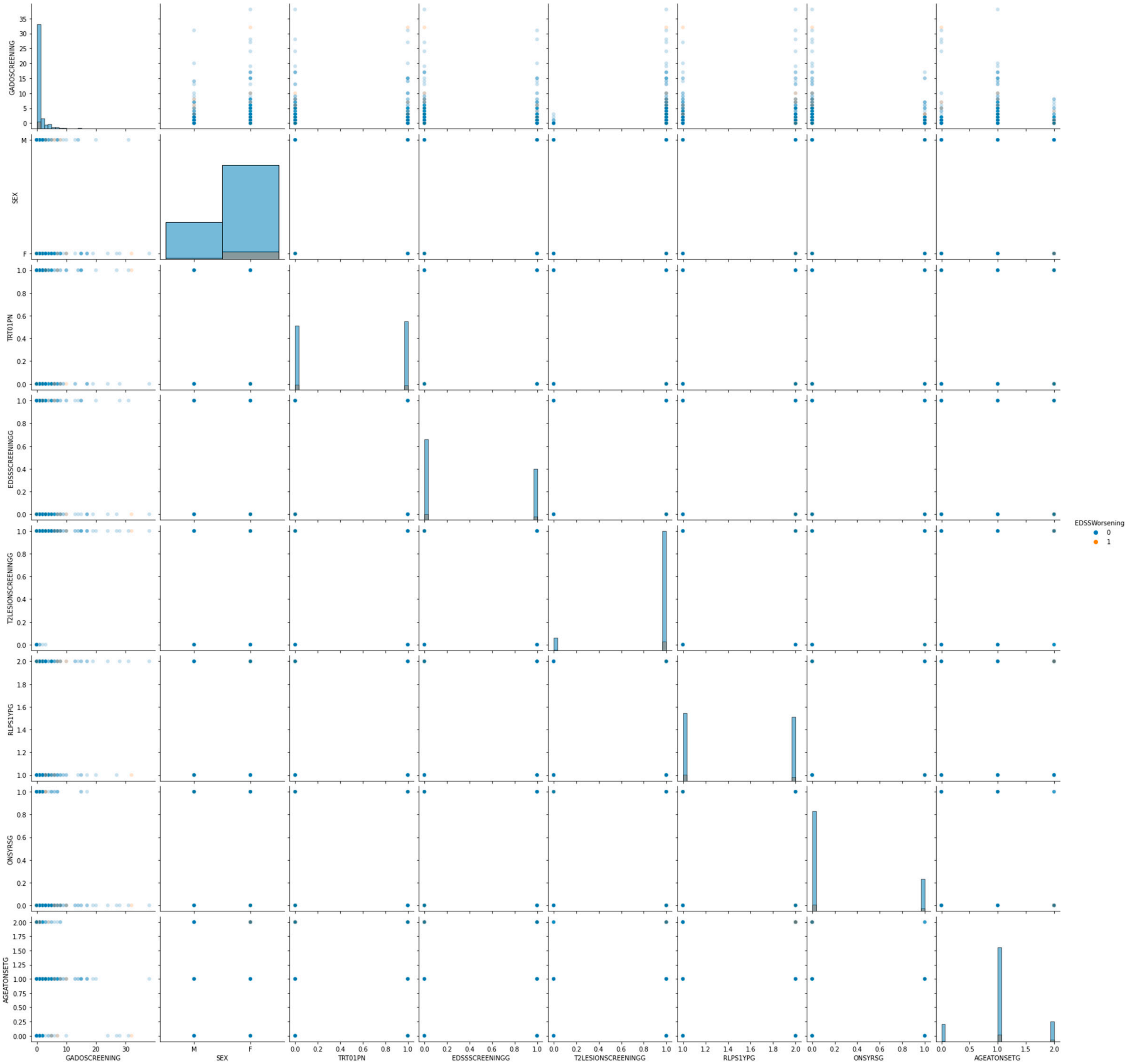

__Figure S2__, Pairwise graph showing how the features used and categorized by the physician differ by EDSS worsening. The diagonal shows the distribution of individual variables for each EDSS worsening class

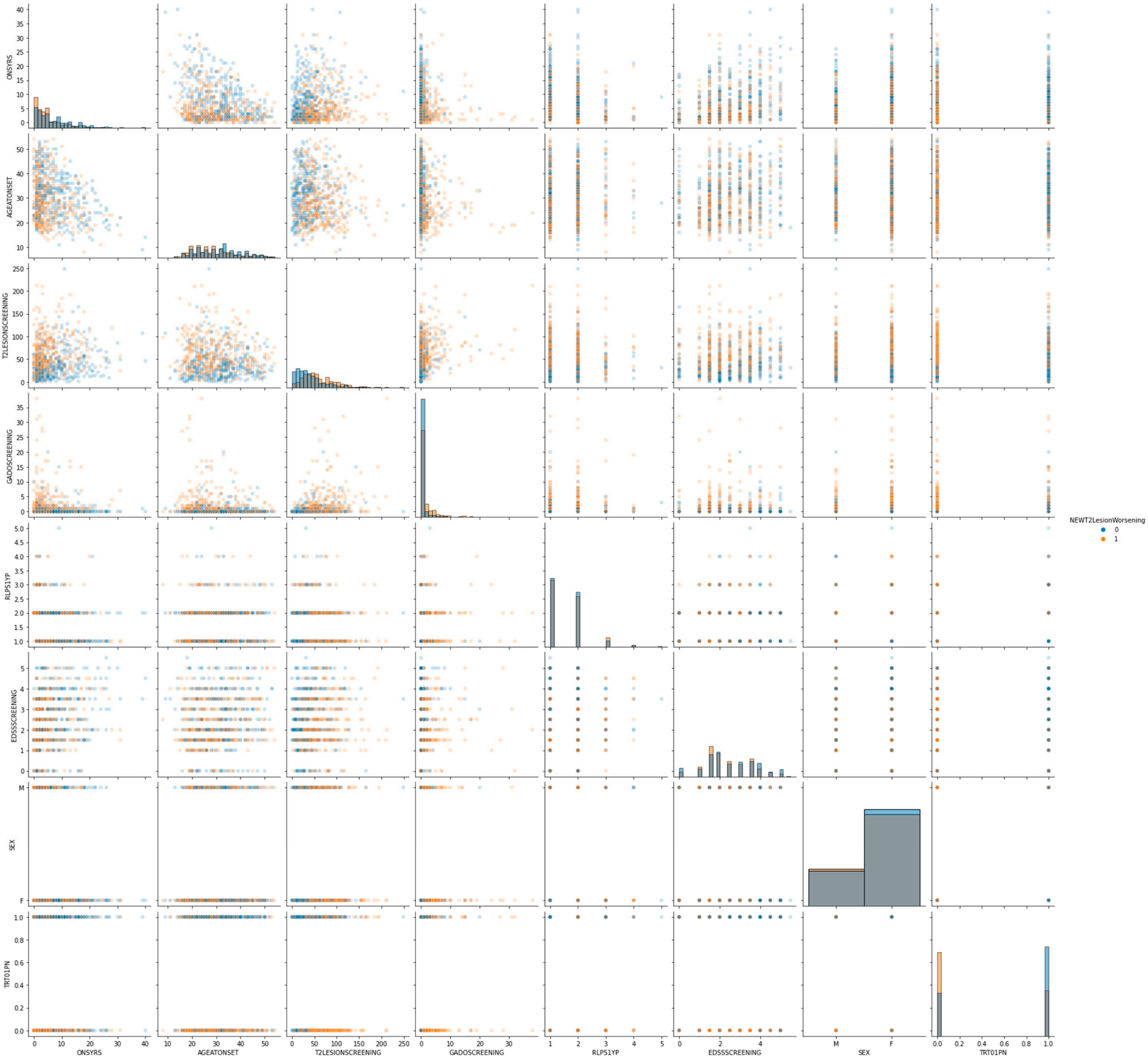

Figure S3, *Pairwise graph showing how the features used by the physician differ by Lesion worsening. The diagonal shows the distribution of individual variables for each Lesion worsening class*

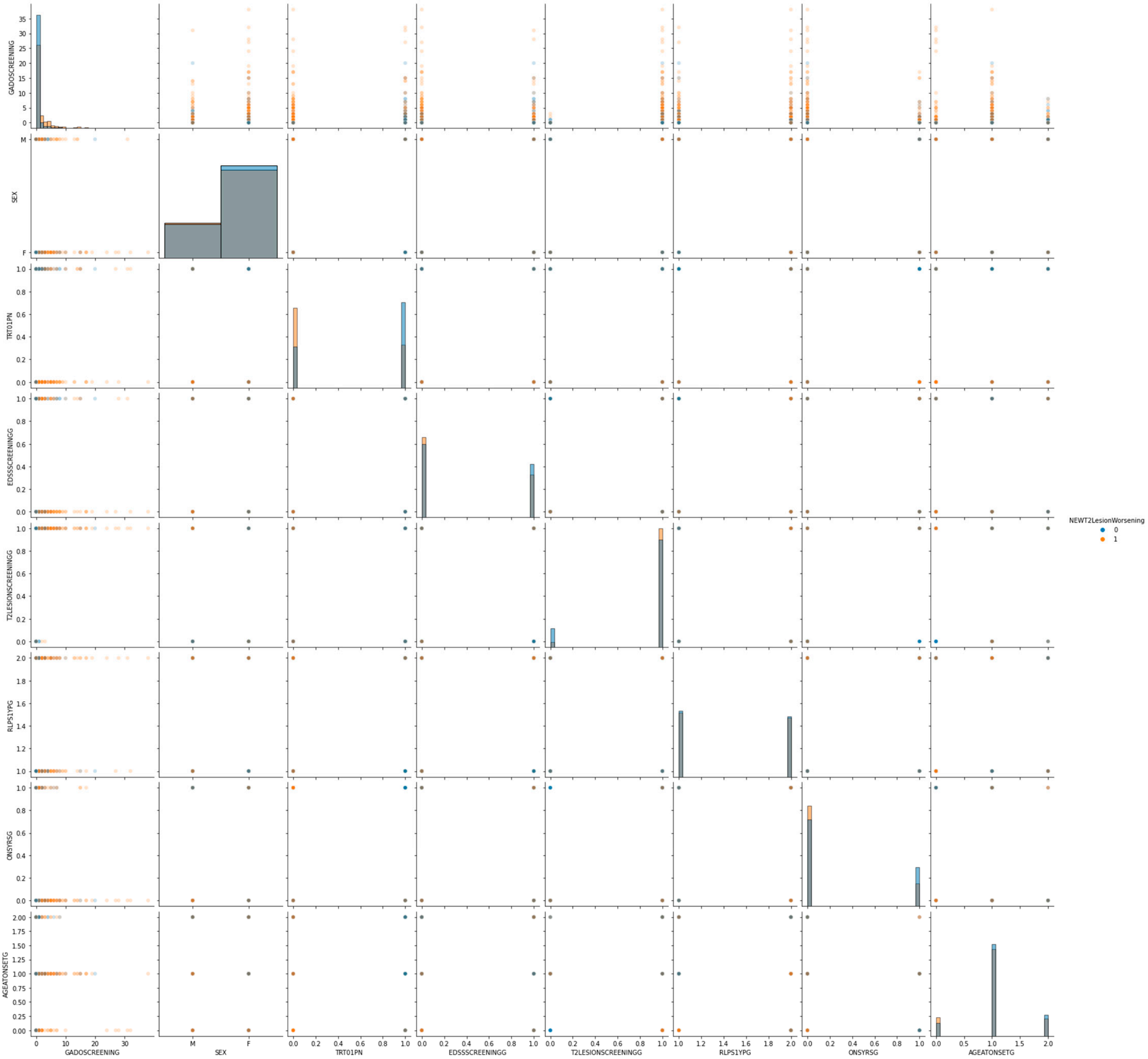

Figure S4, *Pairwise graph showing how the features used and categorized by the physician differ by Lesion worsening. The diagonal shows the distribution of individual variables for each Lesion worsening class*

Table S2, *Performance of models created by the 'All features' method for EDSS worsening prediction*

	AUC score				F1 score				True positive				True negative				False positive				False negative			
	max	min	mean	std	max	min	mean	std	max	min	mean	std	max	min	mean	std	max	min	mean	std	max	min	mean	std
**LogisticRegression**	0.635	0.462	0.548	0.060	0.737	0.698	0.715	0.014	96.0	89.0	91.6	2.417	7.0	3.0	4.8	1.327	7.0	4.0	5.4	1.020	57.0	50.0	54.0	2.366
**DecisionTreeClassifier**	0.548	0.418	0.473	0.043	0.777	0.582	0.677	0.069	108.0	67.0	86.2	14.932	6.0	2.0	3.6	1.625	8.0	4.0	6.6	1.744	79.0	38.0	59.4	14.814
**BaggingClassifier_LogisticRegression**	0.628	0.460	0.532	0.061	0.792	0.751	0.767	0.014	109.0	101.0	104.0	2.828	6.0	2.0	3.6	1.356	8.0	5.0	6.6	1.020	45.0	37.0	41.6	2.653
**BaggingClassifier_DecisionTree**	0.592	0.429	0.491	0.055	0.830	0.755	0.783	0.025	119.0	102.0	109.2	5.636	4.0	1.0	2.4	1.020	9.0	7.0	7.8	0.748	44.0	26.0	36.4	6.020
**AdaBoostClassifier_DecisionTree**	0.513	0.429	0.472	0.030	0.810	0.755	0.780	0.018	116.0	104.0	109.0	4.099	3.0	1.0	2.0	0.632	9.0	7.0	8.2	0.748	41.0	30.0	36.6	3.929
**RandomForestClassifier**	0.600	0.462	0.536	0.048	0.783	0.700	0.732	0.036	107.0	87.0	96.0	8.295	6.0	3.0	4.2	0.980	7.0	4.0	6.0	1.095	58.0	39.0	49.6	8.309
**GradientBoostingClassifier**	0.500	0.486	0.496	0.005	0.905	0.883	0.899	0.008	146.0	141.0	144.4	1.744	0.0	0.0	0.0	0.000	11.0	10.0	10.2	0.400	4.0	0.0	1.2	1.470
**XGBoostClassifier**	0.637	0.427	0.523	0.067	0.815	0.769	0.788	0.016	113.0	105.0	109.4	2.728	5.0	1.0	3.0	1.265	9.0	5.0	7.2	1.327	41.0	33.0	36.2	2.786
**SVCClassifier**	0.672	0.434	0.559	0.081	0.728	0.631	0.671	0.031	94.0	74.0	82.6	6.591	8.0	3.0	5.6	1.744	7.0	2.0	4.6	1.855	72.0	52.0	63.0	6.512
**BaggingClassifier_SVC**	0.500	0.500	0.500	0.000	0.905	0.896	0.903	0.004	146.0	145.0	145.6	0.490	0.0	0.0	0.0	0.000	11.0	10.0	10.2	0.400	0.0	0.0	0.0	0.000
**AdaBoostClassifier_LogisticRegression**	0.615	0.464	0.555	0.054	0.731	0.636	0.709	0.037	95.0	77.0	90.4	6.771	6.0	4.0	5.0	0.894	6.0	4.0	5.2	0.748	69.0	50.0	55.2	7.026
**AdaBoostClassifier_SVC**	0.500	0.500	0.500	0.000	0.905	0.896	0.903	0.004	146.0	145.0	145.6	0.490	0.0	0.0	0.0	0.000	11.0	10.0	10.2	0.400	0.0	0.0	0.0	0.000

Table S3, *Performance of models created by the 'Physician features' method for EDSS worsening prediction*

	AUC score				F1 score				True positive				True negative				False positive				False negative			
	max	min	mean	std	max	min	mean	std	max	min	mean	std	max	min	mean	std	max	min	mean	std	max	min	mean	std
**LogisticRegression**	0.601	0.401	0.509	0.080	0.767	0.743	0.756	0.010	104.0	98.0	101.0	1.897	5.0	1.0	3.2	1.600	9.0	5.0	7.0	1.789	46.0	39.0	42.8	2.227
**DecisionTreeClassifier**	0.682	0.432	0.551	0.082	0.813	0.720	0.766	0.032	110.0	92.0	102.4	7.310	6.0	1.0	4.0	1.789	9.0	4.0	6.2	1.720	51.0	34.0	41.4	7.031
**BaggingClassifier_LogisticRegression**	0.627	0.467	0.553	0.057	0.871	0.826	0.844	0.017	128.0	119.0	122.2	3.187	4.0	1.0	2.6	1.020	9.0	6.0	7.6	1.200	25.0	16.0	21.6	3.137
**BaggingClassifier_DecisionTree**	0.685	0.432	0.552	0.084	0.817	0.720	0.767	0.034	111.0	92.0	102.6	7.526	6.0	1.0	4.0	1.789	9.0	4.0	6.2	1.720	51.0	33.0	41.2	7.250
**AdaBoostClassifier_DecisionTree**	0.543	0.497	0.508	0.018	0.910	0.891	0.902	0.006	144.0	142.0	143.2	0.980	1.0	0.0	0.2	0.400	11.0	9.0	10.0	0.632	2.0	0.0	0.6	0.800
**RandomForestClassifier**	0.572	0.474	0.521	0.035	0.834	0.789	0.817	0.017	122.0	107.0	116.0	5.254	4.0	1.0	2.4	1.020	9.0	6.0	7.8	0.980	37.0	22.0	27.8	5.307
**GradientBoostingClassifier**	0.551	0.448	0.503	0.034	0.879	0.852	0.865	0.010	134.0	128.0	130.6	2.154	2.0	0.0	1.0	0.632	10.0	8.0	9.2	0.748	16.0	10.0	13.2	2.315
**XGBoostClassifier**	0.646	0.449	0.549	0.073	0.825	0.790	0.808	0.011	115.0	106.0	112.8	3.429	5.0	1.0	3.2	1.600	9.0	5.0	7.0	1.673	38.0	29.0	31.0	3.521
**SVCClassifier**	0.614	0.495	0.533	0.048	0.689	0.611	0.662	0.029	86.0	71.0	80.2	5.776	7.0	4.0	5.2	1.166	6.0	3.0	5.0	1.095	73.0	58.0	63.6	5.886
**BaggingClassifier_SVC**	0.500	0.500	0.500	0.000	0.904	0.894	0.902	0.004	144.0	143.0	143.8	0.400	0.0	0.0	0.0	0.000	11.0	10.0	10.2	0.400	0.0	0.0	0.0	0.000
**AdaBoostClassifier_LogisticRegression**	0.637	0.490	0.530	0.055	0.766	0.724	0.748	0.014	103.0	93.0	98.6	3.611	6.0	3.0	3.8	1.166	8.0	4.0	6.4	1.356	51.0	41.0	45.2	3.816
**AdaBoostClassifier_SVC**	0.500	0.500	0.500	0.000	0.904	0.894	0.902	0.004	144.0	143.0	143.8	0.400	0.0	0.0	0.0	0.000	11.0	10.0	10.2	0.400	0.0	0.0	0.0	0.000

Table S4, *Performance of models created by the ' Physician features & classes' method for EDSS worsening prediction*

	AUC score				F1 score				True positive				True negative				False positive				False negative			
	max	min	mean	std	max	min	mean	std	max	min	mean	std	max	min	mean	std	max	min	mean	std	max	min	mean	std
**LogisticRegression**	0.615	0.491	0.561	0.049	0.711	0.626	0.679	0.028	91.0	73.0	84.0	5.933	8.0	4.0	5.6	1.497	6.0	3.0	4.6	1.200	72.0	54.0	61.6	5.817
**DecisionTreeClassifier**	0.625	0.443	0.549	0.063	0.743	0.608	0.683	0.051	98.0	70.0	85.4	10.288	7.0	3.0	5.2	1.327	7.0	3.0	5.0	1.414	76.0	47.0	60.2	10.534
**BaggingClassifier_LogisticRegression**	0.615	0.491	0.561	0.049	0.711	0.626	0.679	0.028	91.0	73.0	84.0	5.933	8.0	4.0	5.6	1.497	6.0	3.0	4.6	1.200	72.0	54.0	61.6	5.817
**BaggingClassifier_DecisionTree**	0.625	0.443	0.549	0.063	0.743	0.614	0.684	0.050	98.0	71.0	85.6	9.992	7.0	3.0	5.2	1.327	7.0	3.0	5.0	1.414	75.0	47.0	60.0	10.237
**AdaBoostClassifier_DecisionTree**	0.577	0.416	0.512	0.066	0.805	0.751	0.774	0.022	113.0	100.0	106.4	4.673	4.0	1.0	3.0	1.265	9.0	6.0	7.2	1.166	46.0	32.0	39.2	4.956
**RandomForestClassifier**	0.660	0.429	0.523	0.080	0.811	0.742	0.776	0.024	116.0	98.0	106.6	6.053	6.0	1.0	3.2	1.720	9.0	4.0	7.0	1.789	48.0	29.0	39.0	6.481
**GradientBoostingClassifier**	0.529	0.483	0.498	0.017	0.898	0.888	0.893	0.004	145.0	140.0	142.0	2.098	1.0	0.0	0.2	0.400	11.0	9.0	10.0	0.632	6.0	0.0	3.6	2.245
**XGBoostClassifier**	0.527	0.471	0.504	0.022	0.740	0.646	0.688	0.036	98.0	79.0	87.0	7.403	5.0	3.0	4.2	0.748	7.0	5.0	6.0	0.632	67.0	47.0	58.6	7.710
**SVCClassifier**	0.692	0.539	0.598	0.055	0.767	0.708	0.745	0.022	102.0	90.0	97.2	4.261	7.0	4.0	5.4	1.020	6.0	3.0	4.8	0.980	56.0	43.0	48.4	4.409
**BaggingClassifier_SVC**	0.500	0.500	0.500	0.000	0.905	0.896	0.903	0.004	146.0	145.0	145.6	0.490	0.0	0.0	0.0	0.000	11.0	10.0	10.2	0.400	0.0	0.0	0.0	0.000
**AdaBoostClassifier_LogisticRegression**	0.622	0.503	0.550	0.045	0.732	0.639	0.690	0.032	94.0	76.0	86.4	6.344	7.0	4.0	5.2	1.166	6.0	4.0	5.0	0.894	69.0	52.0	59.2	6.079
**AdaBoostClassifier_SVC**	0.500	0.500	0.500	0.000	0.905	0.896	0.903	0.004	146.0	145.0	145.6	0.490	0.0	0.0	0.0	0.000	11.0	10.0	10.2	0.400	0.0	0.0	0.0	0.000

Table S5, *Performance of models created by the 'All features' method for Lesion worsening prediction*

	AUC score				F1 score				True positive				True negative				False positive				False negative			
	max	min	mean	std	max	min	mean	std	max	min	mean	std	max	min	mean	std	max	min	mean	std	max	min	mean	std
**LogisticRegression**	0.746	0.662	0.701	0.035	0.746	0.662	0.701	0.035	61.0	48.0	54.2	4.707	55.0	51.0	53.8	1.470	25.0	21.0	22.2	1.470	30.0	17.0	23.8	4.707
**DecisionTreeClassifier**	0.752	0.591	0.687	0.058	0.751	0.591	0.686	0.058	65.0	47.0	54.0	6.573	61.0	44.0	51.8	5.636	32.0	15.0	24.2	5.636	31.0	13.0	24.0	6.573
**BaggingClassifier_LogisticRegression**	0.746	0.669	0.704	0.033	0.746	0.668	0.704	0.033	61.0	49.0	54.8	4.490	55.0	51.0	53.6	1.356	25.0	21.0	22.4	1.356	29.0	17.0	23.2	4.490
**BaggingClassifier_DecisionTree**	0.753	0.642	0.698	0.044	0.753	0.641	0.698	0.044	62.0	56.0	58.4	2.245	56.0	43.0	49.2	4.665	33.0	20.0	26.8	4.665	22.0	16.0	19.6	2.245
**AdaBoostClassifier_DecisionTree**	0.773	0.648	0.714	0.050	0.773	0.647	0.713	0.051	60.0	56.0	58.4	1.625	59.0	43.0	51.6	6.437	33.0	17.0	24.4	6.437	22.0	18.0	19.6	1.625
**RandomForestClassifier**	0.740	0.655	0.712	0.033	0.740	0.655	0.712	0.033	59.0	54.0	56.6	1.855	57.0	47.0	53.0	3.406	29.0	19.0	23.0	3.406	24.0	19.0	21.4	1.855
**GradientBoostingClassifier**	0.760	0.656	0.709	0.042	0.760	0.656	0.709	0.042	60.0	50.0	56.2	3.544	57.0	47.0	53.0	3.795	29.0	19.0	23.0	3.795	28.0	18.0	21.8	3.544
**XGBoostClassifier**	0.779	0.656	0.723	0.051	0.779	0.656	0.723	0.051	61.0	51.0	57.0	4.147	59.0	48.0	54.4	3.929	28.0	17.0	21.6	3.929	27.0	17.0	21.0	4.147
**SVCClassifier**	0.753	0.649	0.713	0.034	0.753	0.649	0.713	0.034	60.0	51.0	55.8	3.059	56.0	49.0	54.0	2.608	27.0	20.0	22.0	2.608	27.0	18.0	22.2	3.059
**BaggingClassifier_SVC**	0.766	0.675	0.722	0.036	0.766	0.675	0.722	0.036	62.0	52.0	57.4	4.128	56.0	52.0	53.8	1.600	24.0	20.0	22.2	1.600	26.0	16.0	20.6	4.128
**AdaBoostClassifier_LogisticRegression**	0.766	0.642	0.708	0.047	0.766	0.642	0.707	0.047	61.0	50.0	56.6	4.224	57.0	45.0	52.4	4.454	31.0	19.0	23.6	4.454	28.0	17.0	21.4	4.224
**AdaBoostClassifier_SVC**	0.760	0.643	0.714	0.051	0.760	0.643	0.714	0.051	61.0	48.0	55.0	5.404	58.0	51.0	55.0	2.757	25.0	18.0	21.0	2.757	30.0	17.0	23.0	5.404

Table S6, *Performance of models created by the 'Physician features' method for Lesion worsening prediction*

	AUC score				F1 score				True positive				True negative				False positive				False negative			
	max	min	mean	std	max	min	mean	std	max	min	mean	std	max	min	mean	std	max	min	mean	std	max	min	mean	std
**LogisticRegression**	0.761	0.699	0.729	0.026	0.761	0.699	0.729	0.026	61.0	54.0	57.8	2.561	60.0	52.0	55.8	2.993	25.0	17.0	21.2	2.993	25.0	18.0	21.0	2.757
**DecisionTreeClassifier**	0.738	0.635	0.701	0.038	0.737	0.634	0.700	0.039	63.0	46.0	54.6	7.003	60.0	51.0	54.6	4.030	26.0	17.0	22.4	4.030	33.0	16.0	24.2	7.194
**BaggingClassifier_LogisticRegression**	0.763	0.686	0.724	0.030	0.763	0.686	0.724	0.030	61.0	53.0	57.6	2.800	60.0	51.0	55.2	3.655	26.0	17.0	21.8	3.655	26.0	18.0	21.2	2.926
**BaggingClassifier_DecisionTree**	0.768	0.659	0.708	0.040	0.768	0.657	0.708	0.041	61.0	55.0	58.8	2.135	59.0	43.0	51.6	5.783	34.0	18.0	25.4	5.783	24.0	18.0	20.0	2.280
**AdaBoostClassifier_DecisionTree**	0.750	0.666	0.706	0.032	0.750	0.666	0.705	0.032	64.0	54.0	58.4	3.611	56.0	48.0	51.6	2.728	29.0	21.0	25.4	2.728	25.0	15.0	20.4	3.555
**RandomForestClassifier**	0.743	0.659	0.707	0.039	0.743	0.658	0.706	0.039	65.0	57.0	59.8	2.993	58.0	44.0	50.4	5.314	33.0	19.0	26.6	5.314	22.0	14.0	19.0	2.828
**GradientBoostingClassifier**	0.749	0.659	0.708	0.033	0.749	0.658	0.708	0.034	65.0	55.0	58.8	3.370	55.0	45.0	51.6	3.666	32.0	22.0	25.4	3.666	24.0	14.0	20.0	3.286
**XGBoostClassifier**	0.750	0.673	0.715	0.030	0.750	0.673	0.714	0.030	64.0	53.0	58.6	3.980	58.0	46.0	52.8	4.534	31.0	19.0	24.2	4.534	26.0	15.0	20.2	3.970
**SVCClassifier**	0.763	0.673	0.724	0.036	0.763	0.673	0.724	0.036	62.0	52.0	57.0	3.406	61.0	50.0	55.8	4.308	27.0	16.0	21.2	4.308	27.0	17.0	21.8	3.429
**BaggingClassifier_SVC**	0.757	0.685	0.724	0.029	0.756	0.685	0.724	0.029	62.0	54.0	58.0	2.608	60.0	50.0	54.8	3.868	27.0	17.0	22.2	3.868	25.0	17.0	20.8	2.713
**AdaBoostClassifier_LogisticRegression**	0.768	0.673	0.720	0.035	0.768	0.673	0.720	0.035	62.0	54.0	57.2	3.059	60.0	50.0	55.0	4.290	27.0	17.0	22.0	4.290	25.0	17.0	21.6	3.200
**AdaBoostClassifier_SVC**	0.761	0.685	0.721	0.030	0.761	0.685	0.721	0.030	63.0	55.0	59.0	3.033	59.0	49.0	53.4	3.720	28.0	18.0	23.6	3.720	24.0	16.0	19.8	3.250

Table S7, *Performance of models created by the 'Physician features & classes' method for Lesion worsening prediction*

	AUC score				F1 score				True positive				True negative				False positive				False negative			
	max	min	mean	std	max	min	mean	std	max	min	mean	std	max	min	mean	std	max	min	mean	std	max	min	mean	std
**LogisticRegression**	0.730	0.634	0.692	0.036	0.730	0.633	0.691	0.036	62.0	54.0	57.4	3.382	54.0	44.0	50.4	3.441	33.0	23.0	26.6	3.441	25.0	17.0	21.4	3.611
**DecisionTreeClassifier**	0.749	0.629	0.699	0.047	0.749	0.627	0.698	0.047	64.0	46.0	57.2	7.985	54.0	49.0	51.8	1.720	28.0	23.0	25.2	1.720	33.0	15.0	21.6	8.139
**BaggingClassifier_LogisticRegression**	0.730	0.634	0.695	0.037	0.730	0.632	0.695	0.037	62.0	55.0	58.0	2.608	54.0	43.0	50.4	3.929	34.0	23.0	26.6	3.929	24.0	17.0	20.8	2.786
**BaggingClassifier_DecisionTree**	0.749	0.640	0.707	0.045	0.749	0.640	0.707	0.045	63.0	53.0	58.6	3.878	55.0	45.0	51.6	3.555	32.0	22.0	25.4	3.555	26.0	16.0	20.2	4.020
**AdaBoostClassifier_DecisionTree**	0.756	0.660	0.699	0.037	0.756	0.660	0.699	0.037	64.0	50.0	57.2	5.115	55.0	48.0	51.8	2.561	29.0	22.0	25.2	2.561	29.0	15.0	21.6	5.276
**RandomForestClassifier**	0.774	0.679	0.725	0.035	0.774	0.679	0.725	0.035	62.0	56.0	59.6	2.939	58.0	50.0	53.4	2.653	27.0	19.0	23.6	2.653	23.0	16.0	19.2	3.124
**GradientBoostingClassifier**	0.743	0.672	0.716	0.030	0.743	0.672	0.716	0.031	63.0	56.0	59.8	2.482	54.0	47.0	51.8	2.638	30.0	23.0	25.2	2.638	23.0	16.0	19.0	2.608
**XGBoostClassifier**	0.742	0.659	0.709	0.032	0.742	0.658	0.709	0.032	62.0	55.0	59.2	2.482	55.0	45.0	51.4	3.499	32.0	22.0	25.6	3.499	24.0	17.0	19.6	2.577
**SVCClassifier**	0.743	0.660	0.702	0.029	0.744	0.660	0.702	0.029	60.0	54.0	57.6	2.332	56.0	49.0	51.8	2.400	28.0	21.0	25.2	2.400	25.0	18.0	21.2	2.561
**BaggingClassifier_SVC**	0.744	0.685	0.707	0.022	0.744	0.685	0.707	0.022	60.0	57.0	58.4	1.020	57.0	49.0	51.8	2.786	28.0	20.0	25.2	2.786	22.0	18.0	20.4	1.356
**AdaBoostClassifier_LogisticRegression**	0.748	0.608	0.684	0.048	0.748	0.608	0.684	0.048	61.0	52.0	56.0	4.147	55.0	43.0	50.6	4.409	34.0	22.0	26.4	4.409	27.0	17.0	22.8	4.400
**AdaBoostClassifier_SVC**	0.742	0.603	0.673	0.045	0.742	0.603	0.673	0.045	59.0	47.0	53.0	4.000	56.0	47.0	51.8	3.600	30.0	21.0	25.2	3.600	32.0	19.0	25.8	4.308
